# Genomic Characterization and Therapeutic Targeting of HPV Undetected Cervical Carcinomas

**DOI:** 10.3390/cancers13184551

**Published:** 2021-09-10

**Authors:** Fiona J. Ruiz, Aishwarya Sundaresan, Jin Zhang, Chandra S. Pedamallu, Mari K. Halle, Vinodh Srinivasasainagendra, Jianqing Zhang, Naoshad Muhammad, Jennifer Stanley, Stephanie Markovina, Hemant K. Tiwari, Perry W. Grigsby, Camilla Krakstad, Julie K. Schwarz, Akinyemi I. Ojesina

**Affiliations:** 1 Division of Biological and Biomedical Sciences Molecular Cell Biology, Washington University School of Medicine, St. Louis, MO 63108, USA; fiona@wustl.edu (F.J.R.); jschwarz@wustl.edu (J.K.S.); 2 Department of Radiation Oncology, Washington University School of Medicine, St. Louis, MO 63108, USA; jin.zhang@wustl.edu (J.Z.); nmuhammad@wustl.edu (N.M.); jastanley@wustl.edu (J.S.); smarkovina@wustl.edu (S.M.); pgrigsby@wustl.edu (P.W.G.); 3 Department of Biostatistics, University of Alabama at Birmingham, Birmingham, AL 35294, USA; aish8586@gmail.com (A.S.); vinodh@uab.edu (V.S.); htiwari@uab.edu (H.K.T.); 4 Institute for Informatics, Washington University School of Medicine, St. Louis, MO 63108, USA; 5 Alvin J. Siteman Cancer Center, Washington University School of Medicine, St. Louis, MO 63108, USA; 6 Department of Epidemiology, University of Alabama at Birmingham, Birmingham, AL 35294, USA; pcs.murali@gmail.com (C.S.P.); mari.halle@uib.no (M.K.H.); jianqing@uab.edu (J.Z.); 7 Department of Obstetrics and Gynaecology, Haukeland University Hospital, 5021 Bergen, Norway; camilla.krakstad@uib.no; 8 Centre for Cancer Biomarkers, Department of Clinical Science, University of Bergen, 5020 Bergen, Norway; 9 O’Neal Comprehensive Cancer Center, University of Alabama at Birmingham, Birmingham, AL 35294, USA; 10 Department of Cell Biology and Physiology, Washington University School of Medicine, St. Louis, MO 63108, USA; 11 HudsonAlpha Institute for Biotechnology, Huntsville, AL 35806, USA

**Keywords:** HPV, palbociclib, cervix

## Abstract

**Simple Summary:**

Persistent HPV infection is a known driver of cervical carcinogenesis, but the existence and biological relevance of HPV undetected (HPV^U^) cervical cancer has been debated. Here we report the results of detailed molecular classification of HPV^U^ cervical cancer, and validate HPV^U^ as a biomarker of poor outcomes. We identify that HPV^U^ cervical cancer tumors harbor mutations affecting cell cycle progression, and in vitro experiments reveal HPV^U^, but not HPV^+^, cells are sensitive to palbociclib monotherapy. HPV^U^ status can be translated into the clinic as a predictive biomarker of poor patient response to standard of care treatments and these patients may benefit from personalized treatment plans. Our results identify palbociclib as a lead candidate as an alternative treatment strategy for HPV^U^ cervical cancer patients. We also suggest that primary cervix tumors be routinely tested for HPV prior to treatment to identify patients who will benefit from more aggressive precision-driven therapy.

**Abstract:**

Cervical cancer tumors with undetectable HPV (HPV^U^) have been underappreciated in clinical decision making. In this study, two independent CC datasets were used to characterize the largest cohort of HPV^U^ tumors to date (HPV^U^ = 35, HPV^+^ = 430). Genomic and transcriptome tumor profiles and patient survival outcomes were compared between HPV^+^ and HPV^U^ tumors. In vitro analyses were done to determine efficacy of the selective CDK4/6 inhibitor palbociclib on HPV^U^ cancer cell lines. Patients with HPV^U^ CC tumors had worse progression-free and overall survival outcomes compared to HPV^+^ patients. *TP53*, *ARID1A*, *PTEN*, *ARID5B*, *CTNNB1*, *CTCF*, and *CCND1* were identified as significantly mutated genes (SMGs) enriched in HPV^U^ tumors, with converging functional roles in cell cycle progression. In vitro HPV^U^, but not HPV^+^, cancer cell lines with wild type *RB1* were sensitive to palbociclib monotherapy. These results indicate that HPV^U^ status can be translated into the clinic as a predictive biomarker of poor patient response to standard of care treatments. We suggest primary cervix tumors be routinely tested for HPV prior to treatment to identify patients who will benefit from more aggressive precision-driven therapy. Our results identify palbociclib as a lead candidate as an alternative treatment strategy for HPV^U^ CC patients.

## 1. Introduction

Cervical cancer (CC) is the fourth leading cause of cancer amongst women worldwide [1]. Persistent human papilloma virus (HPV) infection is causative for at least 90% of cervical cancers, as well as the majority of vulvar, vaginal, anal, penile, and oropharyngeal cancers [2]. Previously published studies have demonstrated that for both oropharyngeal and cervical cancer, patients with undetectable tumor HPV DNA and RNA have worse overall survival outcomes [3,4,5,6,7,8,9,10,11]. Based upon these findings, guidelines now require mandatory HPV testing in all newly diagnosed oropharyngeal squamous cell carcinomas [4].

In the context of cervical cancer, HPV screening is recommended for women aged 30–65 in addition to pap smears every 5 years [12]. However, upon cervical cancer diagnosis there are currently no established guidelines for clinical HPV testing of primary cervix tumors. In clinical practice, most cervical cancer cases are assumed to be HPV-driven. However, retrospective studies suggest that 7–11% of cervical tumors do not have detectable HPV DNA and or RNA, hereafter referred to as HPV undetectable (HPV^U^) tumors [7,8,9,10,13,14]. The Cancer Genome Atlas (TCGA) analysis of cervix cancer also found that 5% of cervical tumors were HPV^U^ [15].

For patients receiving the standard of care concurrent administration of cisplatin chemotherapy with pelvic irradiation [7,8,9,16], HPV^U^ is an indicator for poor progression-free and overall survival. Overall failure rates for standard of care chemoradiation are as high as 67% in some series for patients with HPV^U^ tumors [8]. Identifying HPV^U^ CC before initiating treatment has the potential to improve outcomes by allowing for personalized treatment planning.

In our previous work, we reported on the landscape of genomic alterations in cervical cancer [17], but the sample sizes were inadequate for identifying HPV^U^ associated genomic alterations. To date there have been no comprehensive studies integrating the investigation of genomic characteristics and therapeutic options for HPV^U^ CC compared to HPV^+^ tumors. In this study we aimed to (1) characterize the genomic landscape and transcriptome of HPV^U^ compared to HPV^+^ CC tumors; and (2) investigate clinically actionable targets that are unique to HPV^U^ CC tumors and can be exploited for therapeutic intervention.

## 2. Materials and Methods

### 2.1. Patient Cohorts

**Figure 1:**
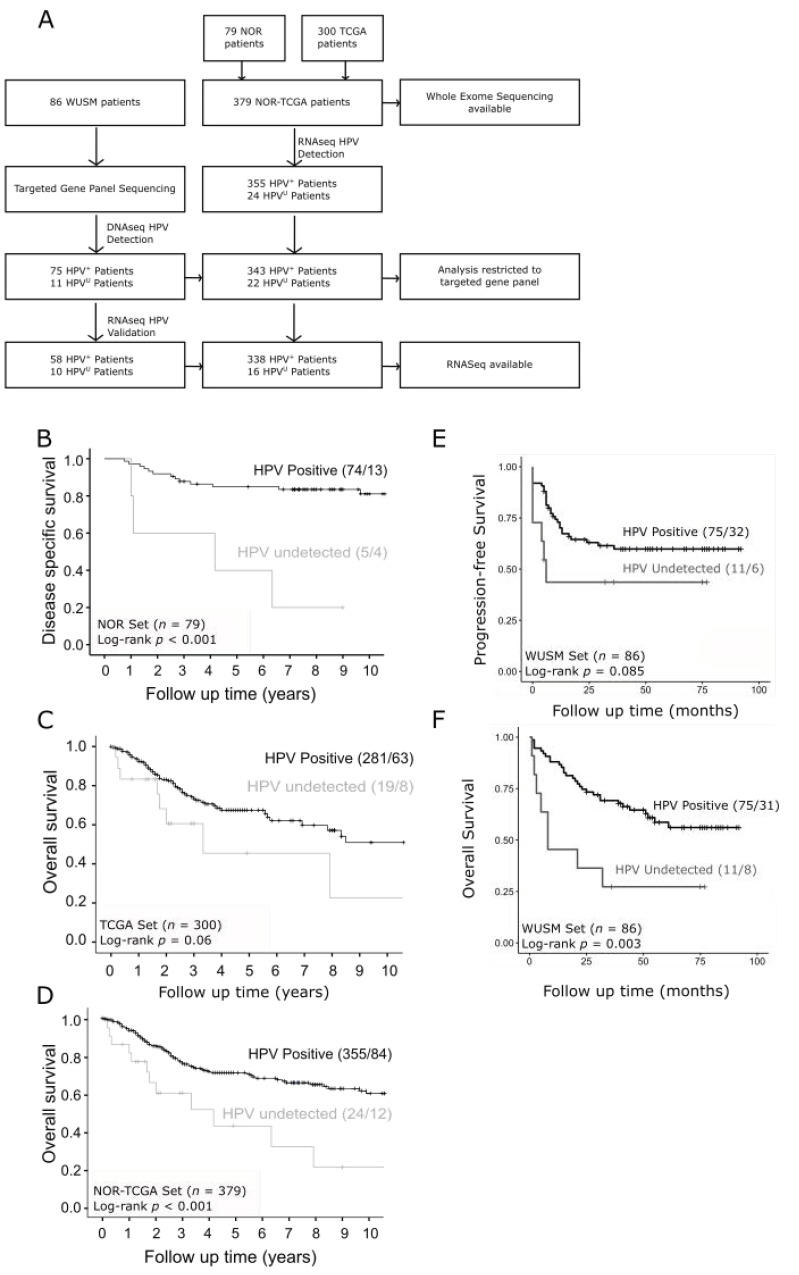
Patient cohorts and survival outcomes in HPV^U^ cervical cancer. (**A**) CONSORT diagram depicting patient samples from NOR-TCGA and WUSM study cohorts and the number of patients at each step of the analysis. (**B**–**E**) shows Kaplan-Meier survival analysis for HPV^U^ (grey) and HPV^+^ (black) patients, using log-rank test to test for significance. (**B**) NOR patients disease specific survival, (**C**) TCGA cervix overall survival and (**D**) overall survival in the combined NOR-TCGA set, (**E**) progression-free survival in WUSM cohort, (**F**) overall survival in WUSM cohort.

Genomic analyses were performed on 2 sets of data (Figure 1A). For the first set, we analyzed whole exome, copy number and RNA sequencing data archived in dbGaP which were originally derived from two published sources: Ojesina et al. [17], and the Cancer Genome Atlas Network (TCGA) [15] (accession numbers phs000600.v1.p1 and phs000178.v11.p8, respectively). The current investigations focused on the prospectively followed Norwegian (NOR) subset of the Ojesina et al. project. The 379 cervical tumors in the “NOR-TCGA set”) included 299 squamous cell carcinomas, 47 adenocarcinomas and 34 other tumors of varying histologies (Table S1), The WUSM cohort included 72 squamous cell carcinomas, 9 adenocarcinomas, and 5 other tumors of varying histologies (Table S1), which were subjected to a targeted exome sequencing panel with 237 genes prioritized based on previous genomics studies [17,18] (Table S2).

### 2.2. Defining Tumors with Undetectable HPV (HPV^U^)

The CESC TCGA standard of HPV detection of at least 1 HPV read per million human reads was employed to identify patients with HPV^+^ positive and HPV^U^ tumors [15]. In the NOR-TCGA set, 24 patient samples met this definition based on PathSeq analyses of RNA sequencing (RNAseq) data [19]. The WUSM cohort defined HPV status using DNA sequencing and validated with RNAseq. Specifically, any sample with no HPV reads detected were defined as HPV^U^ (*n* = 11). These HPV^U^ samples with corresponding RNAseq data (*n* = 10) were then validated by aligning to reference genomes for high-risk HPV 16, 18, 31, 33, 45, 52, 56, 58, and 59. HPV^U^ was defined as any sample with less than 1 HPV read in 1 million human reads. All 10 WUSM samples called as HPV^U^ by DNA probes were also negative using the RNAseq cutoffs.

### 2.3. Genomic DNA Analyses

Mutect2.0 and MutSig2CV [20,21] analyses were performed on the whole-exome sequencing for the 24 HPV^U^ and 355 HPV^+^ tumors in the NOR-TCGA set and significantly mutated genes (SMGs) were identified using a false discovery rate (FDR) of 0.1. SMGs were also analyzed for either co-occurrence or mutual exclusivity of mutations with other SMGs. GISTIC2.0 [22] analyses were also performed on copy number SNP data for the 24 HPV^U^ tumors using FDR ≤ 0.25. Targeted gene exome sequencing for 237 pan cancer associated genes was performed on 86 tumor–blood pairs from the WUSM cohort (Table S2). Combined analysis of NOR-TCGA and WUSM cohort samples were restricted to the 211 genes with mutations in both the whole exome and targeted gene lists.

### 2.4. RNAseq Analysis

RNAseq analyses were performed for 354 NOR-TCGA (73 Norwegian, 281 TCGA) and 68 WUSM cohort tumors independently and used as test and validation datasets, respectively, to assess transcript expression differences between HPV^U^ and HPV^+^ cervical cancer tumors. Mann–Whitney/Wilcoxon tests were used to identify SMGs with differential transcript expression (*p* < 0.05). Additionally, differential expression (DE) analysis was performed using R version 3.5.2 and DESeq2 package on the 10 HPV^U^ and 58 HPV^+^ tumors from the WUSM cohort. DE genes used for subsequent GSEA pathway analysis had a log2 fold change > 1 or < −1 and *p-adj* < 0.01. Pathway analysis was restricted to hallmark gene sets that had at least 10% of the pathway contributed from DE genes and an FDR < 0.01.

### 2.5. Cell Line Selection

Wild-type (WT) RB1 is necessary for palbociclib to be effective [23]. However, the COSMIC cell line database (accessed on November 2019) revealed that the prototypical HPV^U^ cervical cancer cell lines, HT3 and C33A harbor mutant RB1 (Table S4). Therefore, the HPV^U^ Fadu and HPV 16 positive SCC-47 and SCC-154 HNSCC cell lines were used to evaluate efficacy of palbociclib treatment in HPV^+^ and HPV^U^ cancer cell lines. Fadu cells were obtained from the American Type Culture collection (ATCC), SCC-47 from Millipore, and SCC-154 from University of Pittsburgh and maintained in IMDM media (Life Technologies, Carlsbad, CA, USA) with 10% heat inactivated FBS and incubated at 37 °C in 5% CO_2_. Mycoplasma testing was performed periodically to verify no infection. Experiments were performed on cell lines under passage 30. Palbociclib was dissolved in 0.01% of dimethyl sulfoxide (DMSO). All three HNSCC cell lines had WT RB1 genotypes, while Fadu also had overexpression of cyclin D1 as assessed by Western blot using anti-cyclin D1 (92G2) (Cell Signaling Technology, Danvers, MA, USA).

### 2.6. HPV^U^ Cell Line Sensitivity to Palbociclib

Cells were treated with increasing doses of single agent palbociclib (PZ0383) (Sigma-Aldrich, St Louis, MO, USA). Treated cell viability was normalized to untreated control cells. Combination palbociclib and radiation treatment was also evaluated, cells were treated with palbociclib 1hr prior to single-fraction 2 or 4 Gy radiation treatment. Five days after treatment cell viability was quantified using alamarBlue cell viability reagent according to the manufacturer’s protocol (Thermo Fisher Scientific, Waltham, MA, USA).

### 2.7. Palbociclib Induced G1 Arrest and Proliferation Attenuation

G1 cell cycle arrest was evaluated by treating cells with vehicle (0.01% DMSO) or 0.25 µM palbociclib and harvesting them 24 and 48 h after treatment. Cells were stained with propidium iodide and run for flow cytometry on the MACSQuant analyzer (Miltenyl Biotec, Bergisch Gladbach, Germany). Attenuation in proliferation was evaluated by treating cells with either vehicle or 0.25 µM palbociclib and counting cells at 6-, 9-, and 12-days post-treatment. A representative day 12 plate was crystal violet stained and visualized.

### 2.8. Statistics

The Kaplan–Meier method and log rank test were used to determine differences in progression-free and overall survival, using the R version 3.5.2 packages survminer and survival. Fisher exact test was used to identify SMGs in the NOR-TCGA and WUSM cohorts. Mann–Whitney/Wilcoxon tests were used to identify SMGs with differential transcript expression. The Wilcoxon signed-rank test and student t-tests were used for in vitro experiments. *P* less than 0.05 was set as the threshold for significance of all study outcomes.

## 3. Results

### 3.1. Survival Outcomes Are Poor for Patients with HPV^U^ Cervical Tumors

The CONSORT diagram depicting patient/sample distributions are shown in Figure 1A. Patients enrolled in the Norwegian cohort were mostly stage Ib-II and treated by standard of care surgical resection. HPV^U^ patients from this cohort had worse disease-specific survival outcomes compared to HPV^+^ patients (*p* < 0.001) (Figure 1B). The patients enrolled in the TCGA cervix study were not uniformly treated and clinical follow up was not a requirement, despite this we see a similar trend of patients with HPV^U^ cervical tumors having worse overall survival compared to HPV^+^ patients (*p* = 0.06) (Figure 1C). Survival analyses for the combined NOR-TCGA set confirm that patients with HPV^U^ cervix tumors have worse overall survival outcomes compared to those with HPV^+^ cervical cancer (*p* = 0.001) (Figure 1D). Patients from the WUSM cohort had advanced staged disease and were uniformly treated with the standard of care curative intent radiation treatment and concurrent platinum-based chemotherapy (CRT). Similar to other studies, WUSM cohort patients with HPV^U^ cervical tumors had worse progression free survival and higher mortality rates compared to patients with HPV^+^ tumors (Figure 1E,F). Furthermore, across all cohorts HPV^U^ cervical tumors were more likely to have non-squamous histology and occur with higher frequency in patients ≥50 years old compared with HPV^+^ tumors (Table S1).

### 3.2. Discovery of Significantly Mutated Genes Defines a Distinct Biology in HPV^U^ Tumors

Mutect2.0 analyses of the 24 HPV^U^ tumors in the NOR-TCGA set revealed a total of 10,182 somatic mutations, including 6314 missense, 586 nonsense, 2232 silent, 266 splice site mutations, as well as 480 deletions, 90 insertions, and 214 miscellaneous mutations. The aggregate non-silent mutation rate was 3.1 per Mb. MutSig2CV analyses revealed *TP53, PTEN, KRAS*, and *ARID1A* as significantly mutated genes (SMGs) at the false discovery rate of q < 0.1. Recurrent mutations in these genes occurred in 50%, 29%, 21%, and 33% respectively (Table S3).

In order to identify additional putative SMGs associated with HPV^U^ tumors, we compared the relative frequencies of mutations in HPV^U^ versus HPV^+^ cervical tumors, using two complementary datasets (the NOR-TCGA set alone, and a combined NOR-TCGA/WUSM set). For the first set, the comparison of relative mutational frequency was focused on the 1569 genes which met at least one of the following criteria: (i) MutSig2CV *p* value < 0.1 in either HPV^U^ or HPV^+^ tumors; and (ii) any gene with >1 mutation in HPV^U^ tumors and zero mutations in HPV^+^ tumors. These criteria allowed for the investigation of genes which would otherwise be deemed non-significant in MutSig2CV analyses due to the small sample size, with a stringent false discovery rate <0.05 following Fisher’s exact test and Benjamini–Hochberg correction. The genes with higher frequencies of somatic mutations in HPV^U^ compared with HPV^+^ cervical tumors include *TP53, RICTOR, ARID1A, ARHGEF2, ZNF331, CTNNB1, KIAA3012,* and *MC5R* (Table 1). In the second phase, we combined NOR-TCGA and WUSM samples order to increase the sample size for HPV^U^ samples. Comparison of relative mutational frequencies in the combined dataset was focused on the 211 genes with mutations in both the whole exome and targeted gene lists. The genes with significantly higher frequencies in HPV^U^ tumors (Fisher’s exact test with FDR 0.05), were *TP53, ARID5B, ARID1A, PTEN, CTNNB1, CTCF*, and *CCND1* (Table 1). *CCND1* had recurrent P287T mutations.

GISTIC2.0 analysis of somatic copy number alterations (SCNA; threshold of *q* < 0.25) revealed 6 significant focal amplifications in the following cytobands (listed in genomic order with some genes in the peaks in parentheses): 3q26.31 (*GHSR, FNDC3B*), 7p15.3 (*IGF2BP3, IL6*), 8p11.22 (*FGRFR1, TACC1*), 11q13.3 (*CCDN1, FGF3, FGF4*), 11q22.1 (*TRPC6*), 17q12 (*ERBB2*), 19q12 (*CCNE1*), and Xq21.33 (no gene in the peak but close to *FAM133A*) (S4). In addition, six significant focal deletion peaks were identified in cytobands 3p14.2 (*FHIT*), 4q35.2 (*FAT1, CASP3*), 5q12.3 (*PPWD1, RAD17, PIK3R1*), 15q22 (*RNF111, CCNB2*), 19p12 (*RPSAP58*), and 22q13.32 (*PANX2, NCAPH2*) (Figure S1).

**Figure 2:**
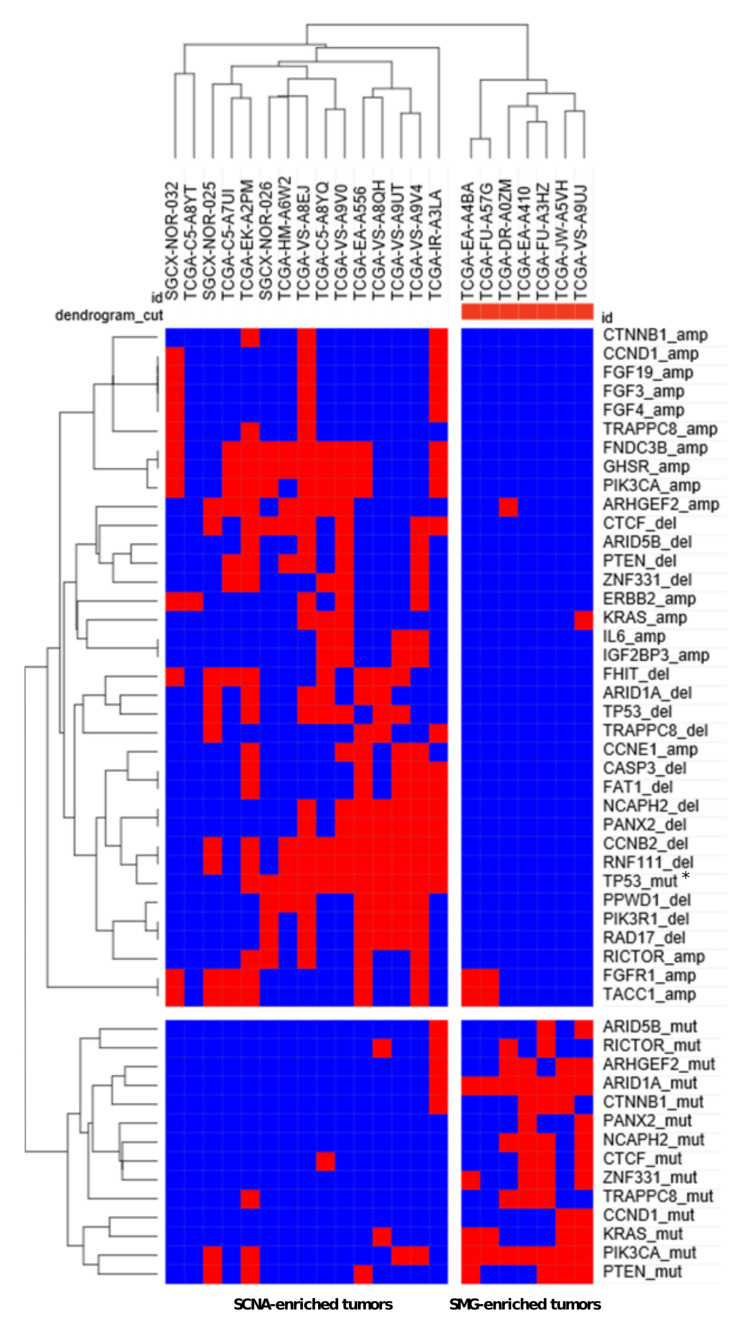
Hierarchical clustering SMGs and SCNAs. A full complement of mutations and SCNAs affecting SMGs and genes in copy number peaks were summarized a matrix and subjected to hierarchical clustering. Red and blue squares represent presence and absence, respectively, of mutation or alteration. Nomenclature: genename_amp, genename_del, and genename_mut represent amplifications, deletions and mutations involving each respective gene. * *p* < 0.05.

Unsupervised hierarchical clustering of a combined set of SMGs and SCNAs yielded a striking segregation of HPV^U^ tumors into two unique subsets: an SMG-rich subset with tumors harboring most of the SMGs except for *TP53*, and an SCNA-rich subset which harbored most of the SCNAs and displayed strong overlap with *TP53* mutation (Figure 2 and Figure S2). On the level of individual SMGs, *ZNF331* mutations co-occurred with *CTCF* (*p* = 0.007623) and *ARID1A* (*p* = 0.00659) mutations, and *KIAA1012* mutations co-occurred with *MC5R* mutations (*p* = 0.000198) (Table S3). *TP53* mutations found in HPV^U^ tumors were mutually exclusive with *ARID1A* (*p* = 0.027), *ZNF331* (*p* = 0.0466), and *NCAPH2* (*p* = 0.0466) mutations (Table S3).

### 3.3. Significantly Mutated Genes Disrupt Cell Cycle Regulation in HPV^U^ Cervical Cancer

**Figure 3:**
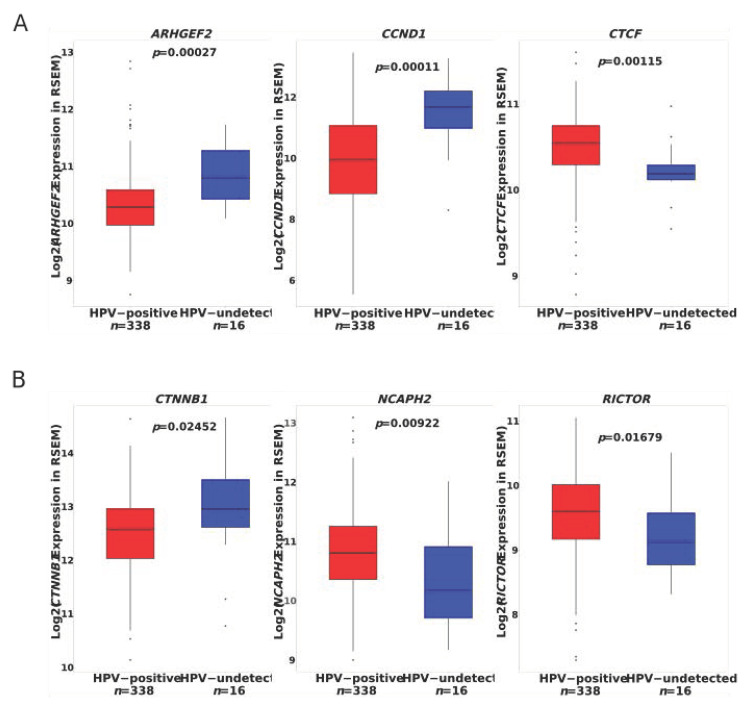
SMGs up- and downregulated in HPV^U^ tumors. (**A**) SMGs in HPV^U^ tumors from the NOR-TCGA patient set that are upregulated and (**B**) down regulated. Mann-Whitney-Wilcoxon *p* values shown.

The SMGs *ARHGEF2, CCND1*, and *CTNNB1* all had higher transcript expression in HPV^U^ tumors from both the NOR-TCGA and WUSM patient cohorts (Figure 3A and Figure S3A). NCAPH2 had lower transcript expression in HPV^U^ tumors in both patient cohorts and CTCF and RICTOR had lower transcript expression in HPV^U^ tumors from the NOR-TCGA set (Figure 3B and Figure S3B). Furthermore, genes found to be differentially expressed between HPV^U^ and HPV^+^ tumors in the WUSM patient cohort were enriched in hallmark E2F targets (FDR < 0.001) and G2M checkpoint pathways (FDR = 0.002347) (Figure S3C–E).

### 3.4. Identification of a Clinically Actionable Target in HPV^U^ CC

**Figure 4:**
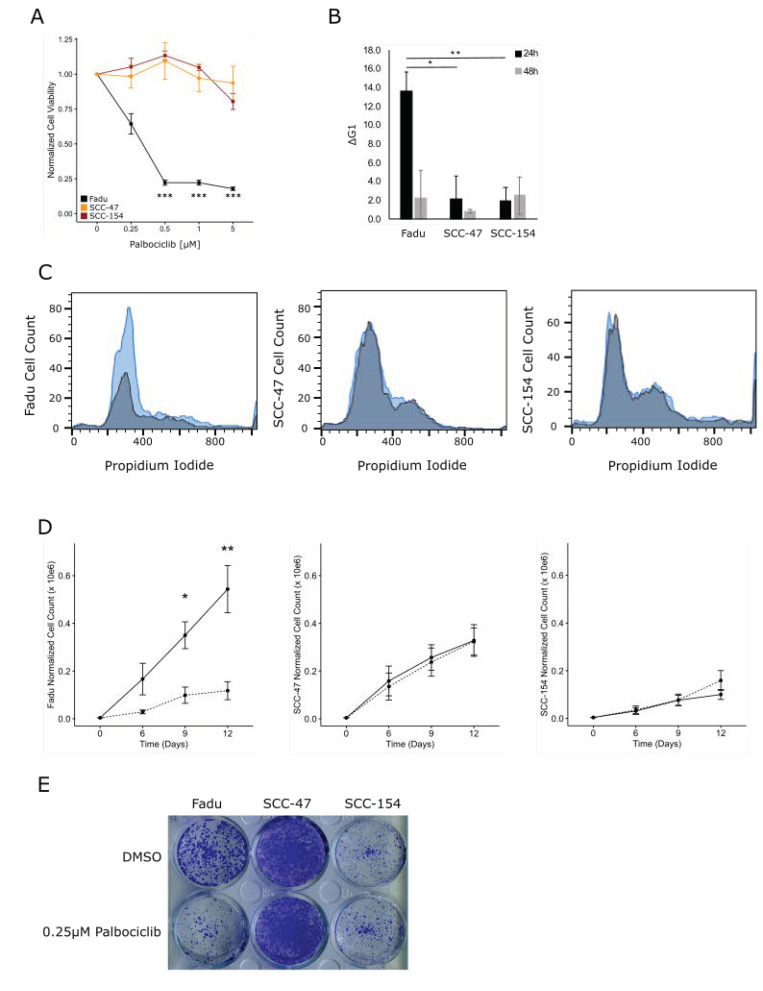
HNSCC cell line sensitivity to Palbociclib. (**A**) Cell viability by alamar blue was evaluated 5 days after Palbociclib treatment alone (Student’s *t*-test, * *p* < 0.05, *** *p* < 0.001). (**B**) Average increase in G1 cell cycle arrest 24 (black) and 48h (grey) after 0.25µM Palbociclib treatment (Avg ± SEM, student’s *t*-test, * *p* < 0.05, ** *p* < 0.01). (**C**) Representative histograms of HPV negative Fadu and HPV positive SCC-47 and SCC-154 (grey = vehicle, blue = 0.25 µM Palbociclib). (**D**,**E**) Cells were counted at 6-, 9-, and 12-days post-Palbociclib treatment and (**D**) proliferation curves after Palbociclib treatment were made (solid = vehicle and dashed = 0.25µM Palbociclib; Wilcoxon test * *p* < 0.05, ** *p* < 0.01). (**E**) Representative crystal violet stained day 12 plate.

All seven SMGs identified in the NOR-TCGA and WUSM combined genomic analysis have converging biological roles in coordinating G1 to S phase cell cycle progression, shown in Figure 4. Additionally, HPV^U^ tumors had higher *CCND1* transcript expression in both the NOR-TCGA and WUSM cohorts, and *CCND1* was present in a significant 11q13.3 amplification peak in the NOR-TCGA set. In HPV^+^ cervical cancer, expression of HPV E7 drives bypass of the G1 cell cycle checkpoint by targeting Rb for proteasomal degradation. Since HPV^U^ cervical cancers may bypass the G1 checkpoint via a different mechanism than HPV^+^ tumors, we hypothesize that HPV^U^ tumors may be more sensitive than HPV^+^ tumors to the FDA approved CDK 4/6 inhibitor palbociclib (Graphical Abstract).

### 3.5. HPV^U^ Cell Lines with Intact RB1 Are Selectively Sensitivity to CDK4/6 Inhibition

Palbociclib restores G1 cell cycle arrest through inhibition of CDK4/6 which attenuates cyclin D1′s ability to phosphorylate and inhibit Rb. HPV^U^ cervical cancer cell lines C33A and HT3 were tested for sensitivity to palbociclib monotherapy alongside the HPV^+^ SiHa and Caski cervical cancer cell lines. We did not observe any sensitivity of either the HPV^U^ or HPV^+^ cell lines to palbociclib (Table S4). Previous groups have established that wild-type (WT) Rb is necessary for palbociclib to be effective, with palbociclib having no effect on proliferation in Rb deficient cell lines even under concentrations more than 50 times greater than that needed for efficacy in Rb proficient cells [23]. Although we did not detect any *RB1* mutations in our HPV^U^ cervical tumor specimens, both HPV^U^ cervical cancer cell lines C33A and HT3 were found to harbor *RB1* mutations according to the COSMIC cell lines database [24] (Figure 4 and Figure S4A). Therefore, in order to evaluate the efficacy of palbociclib treatment in HPV^U^ versus HPV^+^ cell lines we evaluated a panel of head and neck squamous cell carcinoma (HNSCC) cell lines. The HPV^U^ Fadu and HPV 16 positive SCC-47 and SCC-154 HNSCC cell lines all are *RB1* WT, and the Fadu cell line overexpressed cyclin D1 (Figure S4B).

HPV^U^ Fadu cells were sensitive to all doses of palbociclib tested (0.25 µM, *p* < 0.05; 0.5–5 µM, *p* < 0.001) (Figure 4A). Palbociclib had no effect on the HPV^+^ cell line’s viability (Figure 4A). We treated our HNSCC cell line panel with palbociclib 1h prior to radiation treatment and assessed cell viability five days after treatment; we did not see any significant radiosensitization by palbociclib treatment in any of the cell lines tested irrespective of HPV status (Figure S4C).

### 3.6. CDK4/6 Inhibition Induces G1 Arrest and Decreased Proliferation in HPV^U^ Cell Lines

The effect of palbociclib on G1 cell cycle arrest was evaluated by treating cells with vehicle (0.01% DMSO) or 0.25 µM palbociclib and harvesting them 24 and 48 h after treatment. G1 cell cycle arrest was maximal 24 h after palbociclib treatment, with an average increase in G1 cells, across three independent replicates, of 13.6%, 2.2%, and 2.0% for Fadu, SCC-47 and SCC-154 cells respectively (Figure 4B,C). Fadu cells treated with palbociclib had attenuated growth rates compared to vehicle treated controls (D9, *p* < 0.05; D12, *p* < 0.01), while HPV^+^ SCC-47 and SCC-154 showed no delays in growth rate after treatment with palbociclib (Figure 4D,E).

## 4. Discussion

Various methodologies are used to detect HPV in patient tumors, however there is currently no universal consensus on defining HPV^U^ CC. The most prevalent clinical test for HPV is the use of immunohistochemical (IHC) staining of primary tumor biopsies for p16. Although p16 IHC is reproducible and easy to perform in the clinical laboratory setting, p16 expression can occur independently of HPV infection, and may overestimate the number of tumors assumed to be HPV^+^ [25,26]. Since HPV status is associated with patient survival, it is imperative to correctly identify patients with HPV^U^ tumors. In the research setting the current gold standard of HPV testing is RNAseq based detection of HPV viral transcripts [15,17]. Using RNAseq data, researchers align the sequence reads to reference genomes for all HPV genotypes thereby decreasing the amount of false negative calls. However, the threshold of viral transcript expression necessary to define an HPV^+^ tumor varies between research groups. For HNSCC, the TCGA definition of HPV negativity was less than 1000 HPV transcript reads per tumor sample [27]. The TCGA cervix cancer standard for defining HPV negativity was any tumors with less than 1 HPV read per million (RPM) human reads [15]. Future work will need to be done to clarify what threshold of HPV detection is biologically meaningful.

Apart from the limits of detection, it has also been postulated that HPV^U^ tumors may result from the loss of HPV nucleic acids as the tumors become larger and more advanced [13]. Although our study does not rule out this hypothesis, we did not observe any association between tumor stage and HPV^U^ status, suggesting there may be other potential explanations. While the association between HPV infection and cervical cancer is incontestable, there is no evidence to suggest that the cervix is uniquely protected from the non-HPV-related carcinogenic processes that occur in other tissues of the body. In addition, one would intuitively expect that HPV-driven cervical cancer will more likely be diagnosed earlier rather than later in life if HPV detection and treatment are not done at the precancerous stage. Therefore, our confirmation of an association between HPV^U^ tumors and older age of diagnosis supports the possibility that at least some HPV^U^ tumors may indeed not be driven by HPV infection [13].

Similar to previous studies, we identified *TP53, PTEN, ARID1A, ARID5B*, and *CTNNB1* as SMGs in HPV^U^ tumors [14]. Although these findings are strikingly similar to the SMG profile in endometrial carcinomas [28], multiple rounds of histopathological investigations (both published and unpublished) indicate that these tumors were indeed of cervical origin [15,17]. Notably, our novel identification of *CCND1* and *CTCF* as SMGs put the converging role of cell cycle progression in HPV^U^ cervical cancer into clear perspective. In particular, the contribution of *CCND1* was highlighted both by activating P287T mutations and copy number amplifications [29]. It is not surprising to observe that 50% of HPV^U^ tumors harbor *TP53* mutations given the role of the HPV E6 oncogene in abrogating p53 function. It is also intriguing that several novel HPV^U^ associated SMGs have direct and indirect links to HPV biology, suggesting that the effect of the somatic mutations in the absence of HPV may be similar to the synergy between the wild type forms and HPV in cancer. For example, *KIAA1012* encodes TRAPPC8, a transport protein essential for successful HPV cellular entry [30]. The E2 open reading frame of high-risk HPVs possesses a conserved CTCF binding site. Loss of CTCF binding via mutation of the CTCF binding site increases E6 and E7 viral expression with concomitant increase in cellular proliferation [31]. It is tempting to speculate that somatic mutations in human *CTCF* in the absence of HPV may mimic HPV-CTCF interactions and influence tumorigenesis.

The identification of *RICTOR* as an SMG mutation suggests that it may be an oncogene in the context of HPV^U^ tumors with the possibility for therapy with mTORC2 inhibitors. The relatively low expression of *RICTOR* in HPV^U^ tumors is therefore puzzling but may be explained in part by an indirect effect of HPV E6 on RICTOR expression. RICTOR is a target of mir-218 [32], which in turn is known to be suppressed by HPV E6 [33]. Therefore, in the absence of HPV, *RICTOR* expression may be downregulated. Interestingly, a tumor suppressive function has recently been demonstrated for *RICTOR* in p53-mutant medulloblastoma [34]. Some genes in copy number deletion peaks have either known tumor suppressor functions or biological features that support a suppressive role in tumorigenesis. For example, *RNF111* encodes the Arkadia protein which has been shown to enhance tumor suppression in colorectal, renal cell, and lung carcinoma by enhancing TGF-beta signaling [35,36,37], while *PPWD1* encodes a peptidylprolyl isomerase which contains WD40 domains and is thought to play a role in protein folding and pre-mRNA splicing [38,39].

Although our study’s primary focus was in the identification of coding genes differentially mutated and/or expressed in HPV^U^ compared to HPV^+^ CC that contribute to treatment resistance, there is also increasing evidence that micro- and long non-coding RNAs also contribute to disease resistance. One such study found that mir-21 and mir-155 were more highly expressed in CC tumors as compared to normal cervix tissue [40]. Additionally, the study found that the expression of these micro-RNAs differed between HPV^+^ and HPV^−^ CC tumors, however regardless of HPV status both mir-21 and mir-155 were shown to be a significant predictive indicator of cervical cancer. The authors postulated that the expression of these micro-RNAs may be of high diagnostic value as they have the ability to help predict and identify both HPV^+^ and HPV^−^ CC development.

In this study, we are the first to show that HPV^U^ CC tumors harbor mutations and alterations in transcriptional regulation to overcome G1 cell cycle arrest. We identified mutations and/or overexpression of β-catenin and cyclin D1 which binds to CDK4/6 and inhibits Rb and allows cell cycle progression. The FDA approved CDK4/6 inhibitor, palbociclib was evaluated for efficacy as a single agent therapeutic in HPV^U^ and HPV^+^ HNSCC cell lines. In response to single agent palbociclib treatment the HPV^U^ Fadu cell line exhibited increased G1 cell cycle arrest 24 h after treatment, prolonged proliferation attenuation up to 12 days after treatment and decreased cell viability compared to untreated controls. The HPV^+^ SCC-47 and SCC-154 cells showed no significant effects on G1 cell cycle arrest, proliferation rate or cell viability after palbociclib treatment.

A caveat of our present study was that we were unable to assess palbociclib sensitivity using HPV^U^ cervical cancer cell lines due to the C33A and HT3 cell lines harboring RB1 mutations [24], and palbocilib sensitivity has been previously reported to be dependent on intact RB1 signaling. However, our findings using HNSCC cell lines have been corroborated by two recent studies showing that the cervical HPV^U^ cell line C33A is preferentially sensitive to cyclin D1 inhibitors [41,42]. Of note, *RB1* is not frequently mutated in either HPV^U^ or HPV^+^ CC patients from either NOR-TCGA or WUSM cohorts. Additionally, *RB1* mutations have not been identified as frequently mutated in cervix cancer cohorts from other independent studies [15,17]. Therefore, this treatment strategy should still be considered as a viable option for patients with HPV^U^ CC with wild type *RB1*. Lastly, a recent publication found palbociclib was able to sensitize HPV^U^ but not HPV^+^ HNSCC cell lines to radiation treatment [43]. However, in our study, we did not find any radiosensitization when combining palbociclib with radiation treatment. One reason for this discrepancy may be the difference in endpoints used. In our study we used the Alamar Blue cell viability as our endpoint for cell survival, whereas Göttgens et al. used clonogenic potential. It may be that the combination of palbociclib and radiation treatment acts as a cytostatic therapy, preferentially targeting HPV^U^ cancer cells and attenuating their ability to actively engage their cell cycle. Therefore, although combination treatment does not synergize to decrease cell viability, this strategy may be effective to attenuate HPV^U^ cancer cells ability to proliferate. Altogether the results from our study indicate that HPV^U^ cell lines are uniquely responsive to single agent palbociclib treatment and set the stage for clinical testing to determine if patients with HPV^U^ cervical carcinomas could benefit from the addition of palbociclib into their treatment plan.

## 5. Conclusions

HPV status has the potential to be a powerful prognostic biomarker of CC patient progression-free and overall survival. In this multi-institutional study, we have identified and validated HPV^U^ as a poor prognostic marker in cervical cancer. Patients with HPV^U^ cervical tumors had higher recurrence and mortality rates after surgery and standard of care CRT treatment compared to patients with HPV^+^ tumors (Figure 1). In current clinical practice, routine HPV testing is only performed on precancerous cervical lesions; however, our results highlight the need for routine HPV testing of primary cervix tumors in order to identify patients that have HPV^U^ tumors prior to the initiation of treatment. These patients are likely to fail their standard of care treatment and may benefit from alternative treatment plans, which could include radiation or chemotherapy dose escalation, including the addition of biologically targeted agents. In this study, using the largest cohort of HPV^U^ cervical cancers reported to date, we have performed detailed biological characterization which has identified cell cycle regulation as a rational target for HPV^U^ tumors, and, using a panel HPV^+^ and HPV^U^ cell lines, we have shown that the CDK4/6 inhibitor palbociclib is a potential alternative treatment strategy.

## Figures and Tables

**Table 1:** Significantly mutated genes (SMGs) enriched in HPV^U^ tumors.

**Whole-Exome Analysis: 1569 Genes**				
**NOR-TCGA**	**Gene**	**Relative Frequency of HPV Undetected Tumors with Mutation (%)** **(*n* = 24)**	**Relative Frequency of HPV Positive Tumors with Mutation (%)** **(*n* = 355)**	**FDR *p*-Value**
	*TP53*	50.0	3.1	2.65 × 10^−7^
	*ARID1A*	33.3	5.4	2.05 × 10^−2^
	*RICTOR*	20.8	0.6	1.01 × 10^−2^
	*ARHGEF2*	20.8	1.4	3.05 × 10^−2^
	*ZNF331*	16.7	0.3	3.14 × 10^−2^
	*CTNNB1*	16.7	0.3	3.14 × 10^−2^
	*KIAA1012*	16.7	0.3	3.14 × 10^−2^
	*MC5R*	12.5	0	4.41 × 10^−2^
**Combined Targeted Gene Panel and Whole-Exome Analysis: 211 Genes**				
**NOR-TCGA + WUSM**	**Gene**	**Relative Frequency of HPV Undetected Tumors with Mutation (%)** **(*n* = 33)**	**Relative Frequency of HPV Positive Tumors with Mutation (%)** **(*n* = 418)**	**FDR *p*-Value**
	*TP53*	45.45	3.83	8.82 × 10^−9^
	*ARID1A*	33.33	6.22	9.61 × 10^−4^
	*PTEN*	30.3	5.74	1.75 × 10^−3^
	*ARID5B*	21.21	0.72	6.43 × 10^−5^
	*CTNNB1*	15.15	0.96	6.69 × 10^−3^
	*CTCF*	15.15	1.44	1.84 × 10^−2^
	*CCND1*	9.09	0.24	4.11 × 10^−2^

Top: Mutational frequencies of genes with whole exome MutSig2CV *p* value < 0.1, or genes with >1 mutation in HPV^U^ tumors and 0 mutations in HPV^+^ tumors (1569 genes in all) were compared between HPV^U^ and HPV^+^ tumors. Bottom: Mutational frequencies of the 211 genes with mutations observed in both the whole exome and targeted gene lists were compared between HPV^U^ and HPV^+^ tumors.

## Data Availability

NOR-TCGA cohort data archived in dbGaP which were originally derived from two published sources: Ojesina et al. [17], and the Cancer Genome Atlas Network (TCGA) [15] (accession numbers phs000600.v1.p1 and phs000178.v11.p8, respectively). WUSM cohort RNAseq data can be accessed at Gene Expression Omnibus (GEO) with accession number GSE151666.

## Supplementary Materials

The following are available online at https://www.mdpi.com/article/10.3390/cancers13184551/s1, Table S1: Clinical characteristics of patient cohorts; Table S2: Targeted Gene Panel for WUSM cohort sequencing; Table S3: MutSig2CV SMG list and co-occurrence/mutual exclusivity of SMGs in HPV^U^ tumors; Supplementary Table S4: HPV status, RB and *TP53* mutational profile across cell lines; Figure S1: Somatic copy number alterations (SCNAs) in HPV^U^ tumors; Figure S2: Similarity matrix for presence/absence of mutations and SCNAs in HPV^U^ tumors; Figure S3: WUSM SMG transcript expression and DE analysis; Figure S4: CESC and HNSCC cell line sensitivity to Palbociclib mono-therapy and combination with RT.
