# Supplementary material for: Genomic Characterization and Therapeutic Targeting of HPV Undetected Cervical Carcinomas
Source: Cancers (Basel). 2021 Sep 10;13(18):4551. doi: 10.3390/cancers13184551 (PMC8467954; doi:10.3390/cancers13184551)
Supplement: Supplementary file 1 [file cancers-13-04551-s001.zip › RuizF_HPVu_Supp-Figs.pdf]

Table S1: Clinical characteristics of patient cohorts

	TCGA Cohort (*n* = 300)			NOR Cohort (*n*=79)			WUSM Cohort (*n*= 86)		
	HPV Positive	HPV Undetected	*p*-value[a]	HPV Positive	HPV Undetected	*p*-value[a]	HPV Positive	HPV Undetected	*p*-value[a]
**Age at diagnosis**			***p*=0.008**			*p*=0.05			***p*=0.02**
<50	179 (62%)	6 (32%)		58 (78%)	2 (40%)		30 (40%)	0 (0%)	
≥50	108 (38%)	13 (68%)		16 (22%)	3 (60%)		45 (60%)	11 (100%)	
**Histological type**			***p*=0.001**			*p*=0.67			***p*=8.5e-6**
Squamous cell carcinoma	239 (85%)	10 (53%)		46 (62%)	2 (40%)		68 (91%)	4 (36%)	
Adenocarcinoma	39 (14%)	8 (42%)		20 (27%)	2 (40%)		4 (5%)	5 (45%)	
Adenosquamous carcinoma	3 (1%)	1 (5%)		6 (8%)	1 (20%)		2 (3%)	0 (0%)	
Neuroendocrine carcinoma	NA	NA		2 (3%)	0 (0%)		NA	NA	
Small cell	NA	NA		NA	NA		1 (1%)	2 (18%)	
**FIGO stage**			*p*=0.28			*p*=0.27			*p*=0.14
I	151 (55%)	10 (53%)		63 (85%)	3 (60%)		23 (31%)	0 (0%)	
II	62 (23%)	5 (26%)		10 (14%)	2 (40%)		26 (35%)	4 (36%)	
III	44 (16%)	1 (5%)		1 (1%)	0 (0%)		23 (31%)	6 (55%)	
IV	17 (6%)	3 (16%)		0 (0%)	0 (0%)		3 (4%)	1 (9%)	
**Primary Treatment[b]**			NA			*p*=0.61			*p* = 0.52
Radiation +/- chemo	NA	NA		7 (10%)	1 (25%)		74 (99%)	11 (100%)	
Meigs +/- BSO	NA	NA		64 (89%)	3 (75%)		NA	NA	
Hysterectomy + BSO	NA	NA		1 (1%)	0 (0%)		NA	NA	

[a]Pearsons Chi Square test (2-sided)
[b]Missing data for three patients

AARD	CREBBP	FLT4	KMT2C	NFE2L3	SMAD4
ACVR1B	CTCF	FMNL2	KMT2D	NOTCH1	SMARCA2
ACVR2A	CTNNB1	FOXA2	KPTN	NRG2	SMC1A
ADARB1	CXADR	FOXP2	KRAS	NSD1	SOX1
ADRB1	DCAF8L2	GABBR2	KRT10	OLIG1	SOX17
AJUBA	DIAPH1	GATA3	KRT78	OPLAH	SOX8
APC	DNMT3A	GNAS	LIFR	ORAOV1	SOX9
AR	DOCK6	H3F3B	LINC00087	PAXIP1	SPAG1
ARHGAP35	EGFR	HGF	LINC00623	PBRM1	SPOP
ARID1A	EGR3	HIST1H1C	LINC00662	PCBP1	SRRM4
ARID5B	EHMT2	HIST1H2BC	LOC101930221	PDGFRA	STAG2
ASXL1	ELF3	HIST1H2BD	LRRK2	PIK3CA	STK11
ATM	ENSG00000200418	HIST1H2BF	MALAT1	PIK3CG	TAF1
ATR	ENSG00000223804	HIST1H2BL	MAP2K4	PIK3R1	TAF1L
ATRX	ENSG00000233221	HIST1H2BN	MAP3K1	PLEC	TAOK2
ATXN1	ENSG00000253125	HIST1H4D	MAP7	PLEKHG4B	TBP
AXIN2	ENSG00000255498	HIST1H4F	MAPK1	PODNL1	TERF1
B4GALT3	ENSG00000257913	HIST1H4K	MECOM	POLQ	TET2
BAGE2	ENSG00000258430	HIST4H4	MED1	PPAP2B	TGFBR2
BRAF	ENSG00000261405	HLA-A	MED15	PPP2R1A	TLR4
BRCA1	ENSG00000267934	HLA-B	MICA	PRX	TNRC18
BRCA2	ENSG00000269941	HLA-C	MICALCL	PTEN	TNRC6A
BTN2A2	ENSG00000272057	HLA-DRB1	MIR142	PTENP1	TOP1MT
C11orf80	ENSG00000273000	HLA-E	MIR3648	PTPN11	TP53
C9orf91	EP300	HLA-F	MLLT3	RAD21	TPTE
CAPN15	EPHA3	HLA-G	MN1	RB1	TSHZ2
CASP8	EPHB6	HLA-J	MRGPRE	RNA28S5	TSHZ3
CBFB	EPPK1	HLA-L	MT-ND6	RPL5	U2AF1
CBX7	ERBB2	HNRNPUL2	MT-RNR1	RUNX1	USP9X
CCDC97	ERBB3	HOXA9	MTOR	RYR1	VEZF1
CCND1	ERCC2	HRC	MUC2	SETD2	WT1
CD247	ESPNP	HTT	MUC4	SEZ6L2	ZFHX3
CDH1	EZH2	IDH1	MUC5AC	SF3B1	
CDK12	FAM157C	IDH2	MYO15A	SHARPIN	
CDKN1A	FAM194A	IRF2BPL	NANOS1	SHOC2	
CDKN1C	FBXW7	KCNMA1	NAV3	SIGIRR	
CDKN2A	FGFR2	KCNN3	NCOR1	SIN3A	
CEBPA	FGFR3	KCTD1	NCOR1P1	SIX5	
CHEK2P2	FKBP9L	KDM5C	NDUFB2	SLC24A1	
CHGA	FLNA	KDM6A	NF1	SLC2A8	
COL17A1	FLT3	KMT2B	NFE2L2	SMAD2	

Table S2: Targeted Gene Panel for WUSM Cohort Sequencing
Genes included in the WUSM targeted gene panel for next-generation sequencing of patient tumor samples.

Significantly mutated genes (SMGs) in HPV[U] tumors identified by MutSig2CV analysis

**Gene**	**Patients**	**Relative frequency**	**Unique sites**	**Non-silent mutations**	**Missense mutations**	**Silent mutations**	**"LOF" mutations**	**FDR**
TP53	12	50%	13	13	8	1	5	9.03E-06
PTEN	7	29%	9	11	6	0	5	5.27E-04
KRAS	5	21%	3	5	5	0	0	1.28E-03
ARID1A	8	33%	12	12	3	0	9	1.74E-02

Co-occurrence of SMGs in HPV[U] tumors

	ZNF331 WT	ZNF331 Mutant	*p*-value
CTCF WT	19	1	7.623E-03
CTCF Mutant	1	3	

	ZNF331 WT	ZNF331 Mutant	*p*-value
ARID1A WT	16	0	6.59E-03
ARID1A Mutant	4	4	

	KIAA1012 WT	KIAA1012 Mutant	*p*-value
MC5R WT	20	1	1.98E-04
MC5R Mutant	0	3	

Mutual exclusivity of SMGs in HPV[U] tumors

	TP53 WT	TP53 Mutant	*p*-value
ARID1A WT	5	11	2.7E-02
ARID1A Mutant	7	1	

	TP53 WT	TP53 Mutant	*p*-value
ZNF331 WT	8	12	4.66E-02[1]
ZNF331 Mutant	4	0	

	TP53 WT	TP53 Mutant	*p*-value
NCAPH2 WT	8	12	4.66E-02[1]
NCAPH2 Mutant	4	0	

Table S3: MutSig2CV SMG list and co-occurrence/mutual exclusivity of SMGs in HPV[U] tumors
"LOF" depicts nonsense, frameshift or splice-site mutations (which are often regarded as "loss of function" mutations; FDR depicts false discovery rate (Benjamini-Hochberg procedure, [1]one-tailed test).

Tissue type	Cell Line	HPV status	RB1	TP53
CESC	C33A	-	c.1961-1G>A	p.R273C
	HT3	-	p. Q444R	p.G152V/p.G245V/p.E 285K
	CaSki	HPV 16	WT	WT
	SiHa	HPV 16	WT	WT
HNSCC	Fadu	-	WT	p.R248L
	UM-SCC-47	HPV 16	WT	WT
	UPC1-SCC-154	HPV 16	WT	WT

Table S4: HPV status, RB and TP53 mutational profile across cell lines
COSMIC cell lines database used to identify RB1 and TP53 genetic alterations.

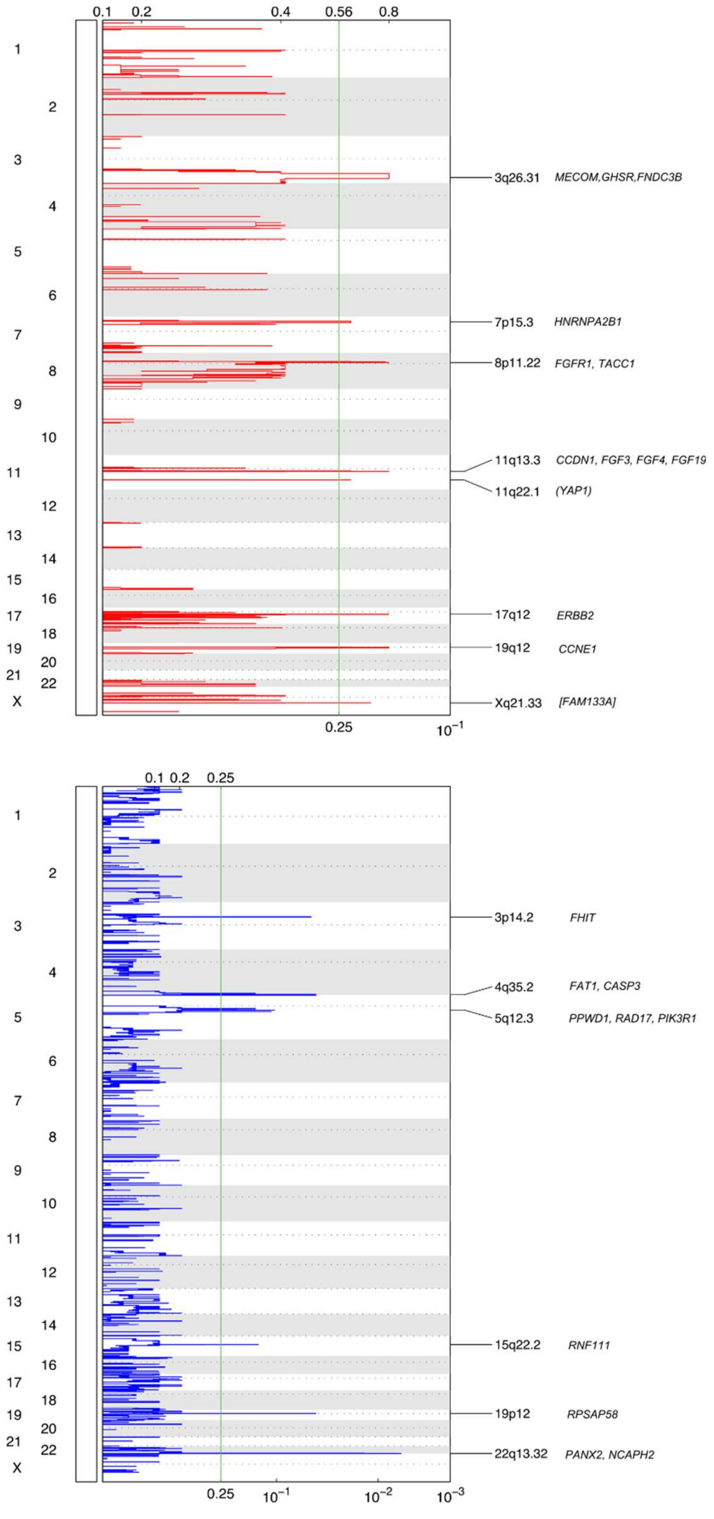

Figure S1: Somatic copy number alterations (SCNAs) in HPV^U tumors

GISTIC2.0 analysis of A) amplifications and B) deletions in 24 HPV^U tumors (threshold of $q < 0.25$).

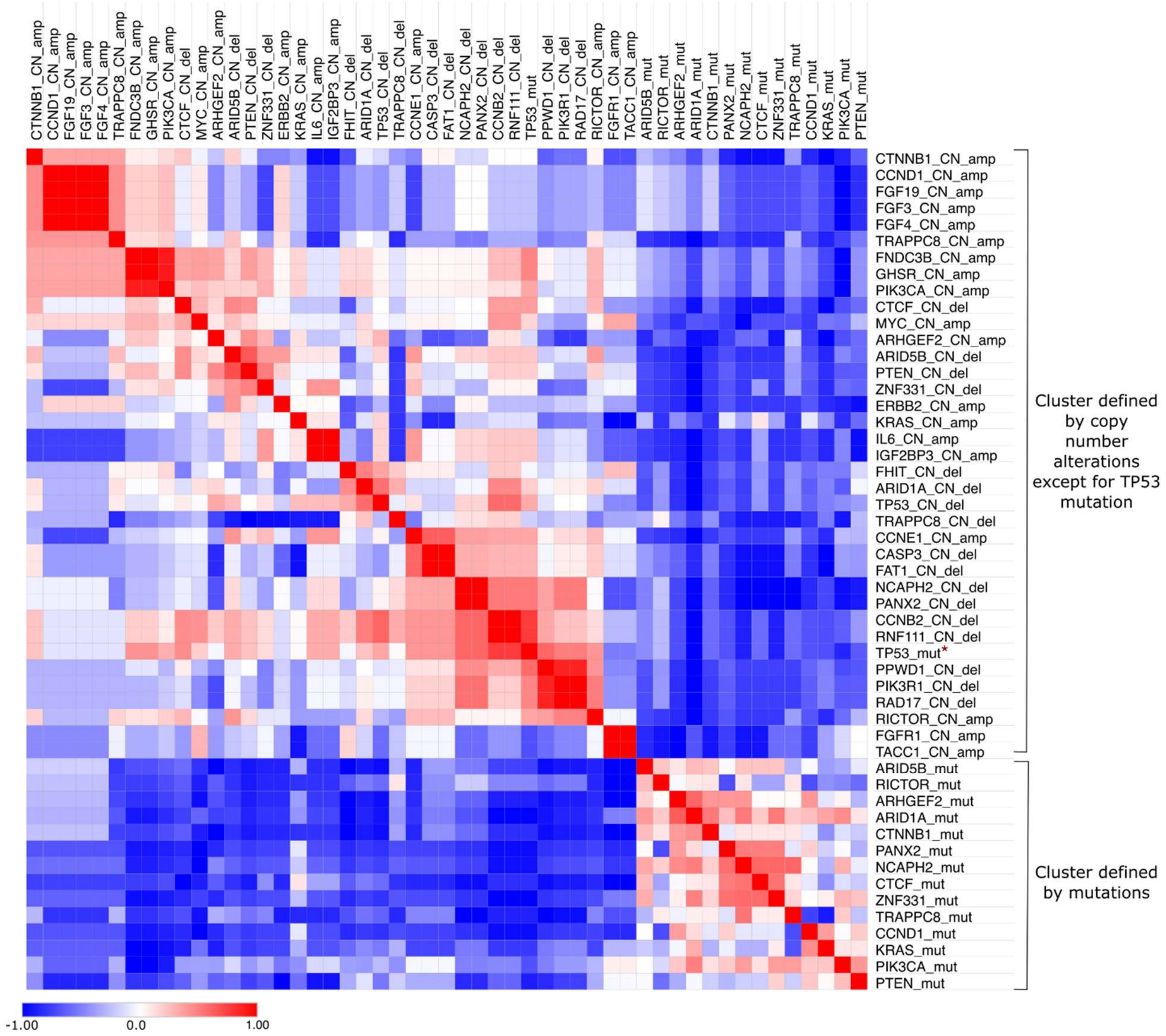

Figure S2: Similarity matrix for presence/absence of mutations and SCNAs in HPV[U] tumors

A complementary depiction of data presented in Main Figure 2. Pearson correlation-based similarity heatmap was generated on data for presence or absence of mutations and SCNAs using the Broad Morpheus suite of tools.

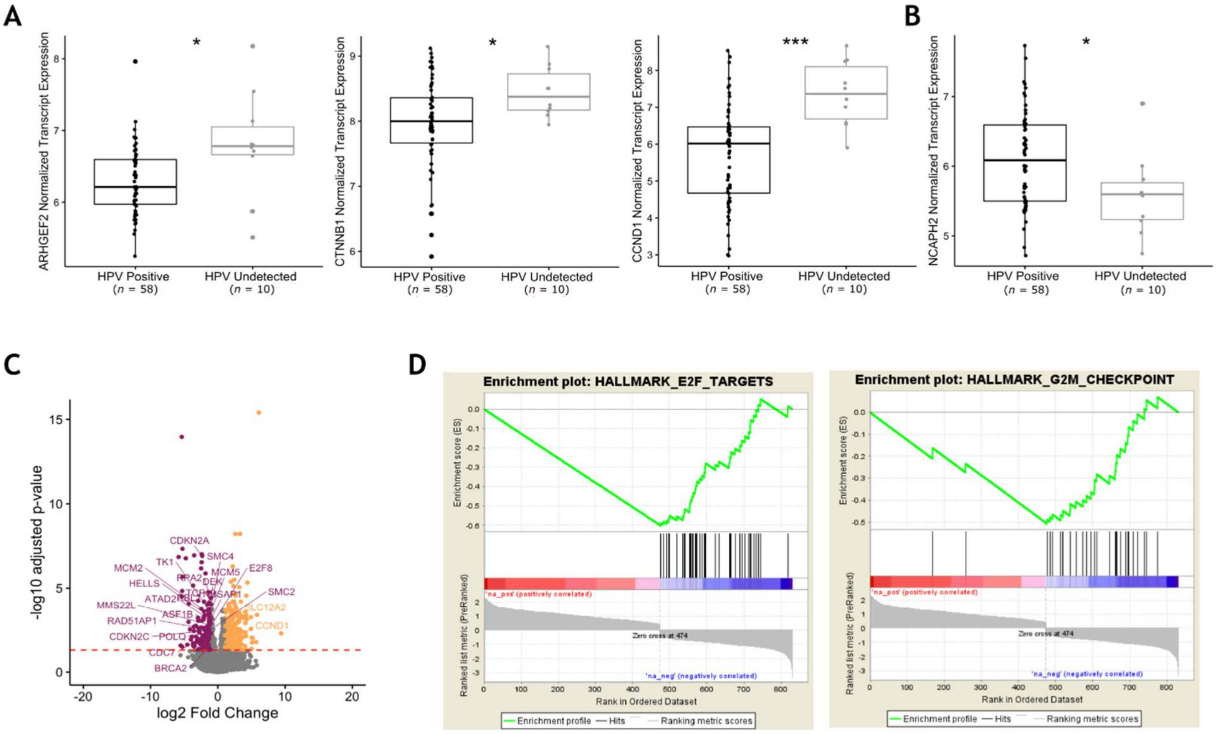

E

Hallmark Pathway	DE Genes	% of pathway DE Genes	ES	NES	NOM *p*-val	FDR *q*-val	FWER *p*-val	RANK AT MAX
E2F TARGETS	39	19.5	-0.60101	-3.43487	< 0.001	< 0.001	< 0.001	356
G2M CHECKPOINT	29	14.5	-0.5064	-2.5845	< 0.001	0.002	0.001	355

Figure S3: WUSM SMG transcript expression and DE analysis

A) SMGs in HPV undetected tumors from the WUSM patient cohort that are upregulated and B) down regulated (Wilcoxon test $*p < 0.05$, $***p < 0.001$). Differentially expressed genes with log2 fold change > 1 or < -1 and *p*-value < 0.05 were used for GSEA pathway analysis. C) Volcano plot of log2 fold change vs -log10 *p*-value for differentially expressed genes in RNAseq library (yellow = DE > 1, purple = DE < -1); gene labels indicate the significant DE genes from the hallmark E2F targets and G2M checkpoint pathways. D) Significant pathways altered in HPV^U vs HPV^+ patient tumors. E) Table of significantly altered pathways from GSEA analysis.

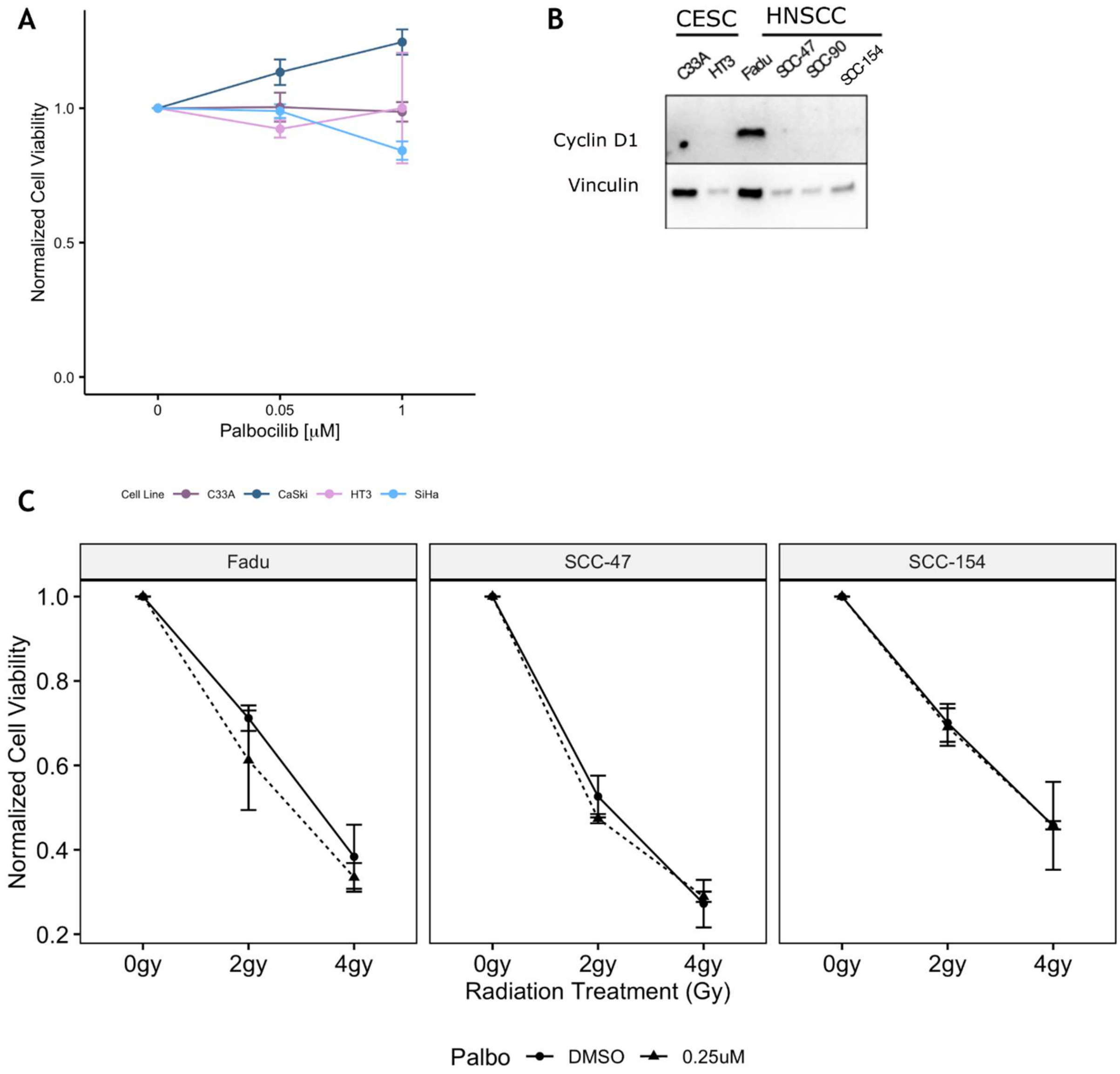

Figure S4: CESC and HNSCC cell line sensitivity to Palbociclib mono-therapy and combination with RT
A) Cervical cancer cell line sensitivity to palbociclib monotherapy (HPVU: C33A, HT3; HPV+: CaSki, SiHa). B) Cyclin D1 protein expression by western blot analysis (vinculin serves as a loading control). C) Cell viability by alamar blue was evaluated 5 days after Palbociclib in combination with radiation treatment (Student's t-test, no significance).
